# Association of Neighborhood Deprivation With Epigenetic Aging Using 4 Clock Metrics

**DOI:** 10.1001/jamanetworkopen.2020.24329

**Published:** 2020-11-04

**Authors:** Kaitlyn G. Lawrence, Jacob K. Kresovich, Katie M. O’Brien, Thanh T. Hoang, Zongli Xu, Jack A. Taylor, Dale P. Sandler

**Affiliations:** 1 Epidemiology Branch, National Institute of Environmental Health Sciences, National Institutes of Health, Research Triangle Park, North Carolina; 2 Epigenetic and Stem Cell Biology Laboratory, National Institute of Environmental Health Sciences, National Institutes of Health, Research Triangle Park, North Carolina

## Abstract

**Importance:**

Neighborhood deprivation is associated with age-related disease, mortality, and reduced life expectancy. However, biological pathways underlying these associations are not well understood.

**Objective:**

To evaluate the association between neighborhood deprivation and epigenetic measures of age acceleration and genome-wide methylation.

**Design, Setting, and Participants:**

This cross-sectional study used data from the Sister Study, a prospective cohort study comprising 50 884 women living in the US and Puerto Rico aged 35 to 74 years at enrollment who had a sister with breast cancer but had not had breast cancer themselves. Cohort enrollment occurred between July 2003 and March 2009. Participants completed a computer-assisted telephone interview on demographic, socioeconomic, lifestyle, and residential factors and provided anthropometric measures and peripheral blood samples at a home examination. DNA methylation data obtained for 2630 non-Hispanic White women selected for a case-cohort study in 2014 were used in this cross-sectional analysis. DNA methylation was measured using the HumanMethylation450 BeadChips in whole blood samples collected at baseline. Data analysis for this study was performed from October 17, 2019, to August 27, 2020.

**Exposures:**

Each participants’ primary address was linked to an established index of neighborhood deprivation.

**Main Outcomes and Measures:**

Epigenetic age was estimated using 4 epigenetic clocks (Horvath, Hannum, PhenoAge, and GrimAge). Age acceleration was determined using residuals from regressing chronologic age on each of the 4 epigenetic age metrics. Linear regression was used to estimate associations between neighborhood deprivation and epigenetic age acceleration as well as DNA methylation at individual cytosine-guanine sites across the genome.

**Results:**

Mean (SD) age of the 2630 participants was 56.9 (8.7) years. Those with the greatest (>75th percentile) vs least (≤25th percentile) neighborhood deprivation had higher epigenetic age acceleration estimated by Hannum (β = 0.23; 95% CI, 0.01-0.45), PhenoAge (β = 0.28; 95% CI, 0.06-.50), and GrimAge (β = 0.37; 95% CI, 0.12-0.62). Increasing US quartiles of neighborhood deprivation exhibited a trend with Hannum, PhenoAge, and GrimAge. For example, GrimAge showed a significant dose-response (*P* test for trend <.001) as follows: level 2 vs level 1 (β = 0.30; 95% CI, 0.17-0.42), level 3 vs level 1 (β = 0.35; 95% CI, 0.19-0.50), and level 4 vs level 1 (β = 0.37; 95% CI, 0.12-0.62). Neighborhood deprivation was found to be associated with 3 cytosine-phosphate-guanine sites, with 1 of these annotated to a known gene *MAOB* (*P* = 9.71 × 10^−08^).

**Conclusions and Relevance:**

The findings of this study suggest that residing in a neighborhood with a higher deprivation index appears to be reflected by methylation-based markers of aging.

## Key Points

**Question:**

Is living in a neighborhood with a high concentration of household and population features characteristic of lower socioeconomic status (ie, a neighborhood with high deprivation) associated with epigenetic age acceleration across first- and second-generation clock metrics?

**Findings:**

This cross-sectional study comprised 2630 women who had a sister with breast cancer but had not had breast cancer themselves. Those living in areas with the greatest compared with least neighborhood deprivation had higher epigenetic age acceleration estimated by Hannum, PhenoAge, and GrimAge clocks but not the Horvath clock.

**Meaning:**

The results of this study suggest that residing in a neighborhood with a higher deprivation index appears to be reflected by methylation-based markers of aging.

## Teaser

This cross-sectional study examines the association between neighborhood deprivation and estimates of epigenetic age acceleration and genome-wide methylation among women in the US and Puerto Rico.

## Introduction

Research suggests widening socioeconomic disparities in life expectancy in the US.^1,2^ Socioeconomic status (SES) is a composite of an individual’s economic and sociologic standing that is measured at both the individual and neighborhood levels. Both individual-level and small-area SES factors, such as living in a socioeconomically disadvantaged neighborhood, are independently associated with chronic disease morbidity and mortality. Low neighborhood-level SES, or neighborhood deprivation, is the concentration of disadvantage associated with social and physical disorder.^3^ Neighborhood deprivation may increase the risk of early onset of age-related disease,^4,5^ reduced life expectancy,^6^ and all-cause mortality.^7^

Mechanisms linking neighborhood deprivation and age-related disease are multifactorial and complex.^8^ Residence in a deprived neighborhood can lead to poor health behaviors,^9,10^ increased toxicant exposures,^11^ lack of access to health services,^12^ and low social cohesion.^13^ Living in a deprived neighborhood can also lead to chronic stress^8,14,15,16^ and induce biological weathering in endocrine and inflammatory systems. Markers of biological aging, such as higher allostatic load^16^ and reduced telomere length,^17^ are linked with greater neighborhood deprivation.

Emerging evidence suggests that epigenetics may be implicated in biological mechanisms underpinning this association.^18,19^ Epigenetic processes are malleable changes in gene expression not due to alterations in the underlying genetic code (DNA sequence). DNA methylation is the most well-understood epigenetic factor and involves the addition of a methyl group to DNA, typically at the 5-carbon of cytosine at cytosine-phosphate-guanine (CpG) dinucleotides. Recently developed epigenetic clocks are a class of biological age estimators that use DNA methylation at predetermined CpG sites to estimate biological variation among those with the same chronologic age. These clocks may be a more sensitive measure of biological aging and are better at estimating chronologic age than other markers, including telomere length.^20^ First-generation clocks developed by Horvath^21^ and Hannum et al^22^ used chronologic age to identify age-related CpG sites, while second-generation clocks, PhenoAge^23^ and GrimAge,^24^ incorporated other phenotypic information to inform CpGs used in their clock metrics. PhenoAge and GrimAge clocks have been demonstrated to be superior to first-generation clocks in estimating mortality (ie, life span),^25^ with GrimAge as the best estimator of life span.

Prior epigenome-wide association studies have demonstrated links between neighborhood deprivation measures and methylation at specific CpG sites in blood, including loci annotated to genes associated with stress and inflammatory response.^18^ Other studies have identified differential expression of inflammatory pathway–related genes associated with adverse social conditions.^26^ These data suggest that epigenetic factors may be implicated in how neighborhood exposures impact biological health and that these epigenetic factors may be viable markers of residential neighborhood traits. However, few data exist on the association between neighborhood deprivation and epigenetic aging.

Only a small number of CpGs are included in the clocks’ metrics. The CpGs are intended as markers of aging (patterns of CpGs are associated with the outcome) but are not exhaustive listings of the specific individual CpGs that might be associated with either exposures or outcomes related to aging. While CpG clocks developed in surrogate tissues, such as blood, have the advantage of being promising environmental biomarkers of exposure, epigenome-wide approaches provide the opportunity to identify novel associations in many different genes.^27^ We therefore sought first to assess neighborhood deprivation in association with epigenetic age acceleration using different epigenetic clocks and second to test the association between neighborhood deprivation and DNA methylation across the genome.

## Methods

The Sister Study is a prospective cohort study comprising 50 884 women living in the US and Puerto Rico aged 35 to 74 years at enrollment who had a sister with breast cancer but had not had breast cancer themselves (2003-2009).^27^ Participants completed a computer-assisted telephone interview on demographic, socioeconomic, lifestyle, and residential factors and provided anthropometric measures and peripheral blood samples at a home examination. A subset of White non-Hispanic participants living in the US with an available blood sample were selected for a case-cohort study (July 17, 2014) designed to assess the association between DNA methylation and incident breast cancer (n = 2878). At the time of case-cohort selection, 1542 women had developed breast cancer and 1336 women were randomly selected from the full cohort. From October 17, 2019, to August 27, 2020, we used data from these case-cohort participants to evaluate associations of neighborhood deprivation and methylation-based measures in a cross-sectional study. We excluded participants with low-quality DNA methylation (n = 102), missing neighborhood deprivation values (n = 5), covariate information (n = 133), or sampling weight (n = 2), resulting in an analytic sample of 2630. The Sister Study was approved by the National Institute of Environmental Health Sciences and Copernicus Group institutional review boards. Participants provided written informed consent; there was no financial compensation. This study followed the Strengthening the Reporting of Observational Studies in Epidemiology (STROBE) reporting guideline for observational studies.

Neighborhood deprivation was defined using the Area Deprivation Index (ADI), a census-based socioeconomic index developed by Kind et al.^28^ The ADI uses information from SES domains of income, education, employment, and housing quality obtained from the American Community Survey. This index is calculated from 17 census indicators that are multiplied by previously published factor weights^29^ and summed for each block-group and then transformed into a standardized index with a mean (SD) of 100 (20). The ADI assigns ranked percentiles that range from 1 to 100, where 1 represents the least deprivation. We linked the 2000 ADI to each participant’s geocoded residential address at enrollment (2003-2009) using block-group Census ID (Federal Information Processing Standards code). We assessed the ADI as a categorical variable defined by US percentiles of neighborhood deprivation (level 1: ≤25th US percentile, level 2: >25th-≤50th percentile, level 3: >50th-75th percentile, and level 4: >75th percentile).

Preprocessing of peripheral blood collected at enrollment for DNA methylation measurement is detailed elsewhere.^27,30^ Briefly, DNA methylation was assessed using the HumanMethylation450 BeadChips (Illumina Inc). Data preprocessing and quality control were completed using the ENmix R package.^31^ This process included the following steps: reducing background noise with ENmix background correction, regression on logarithm of internal control probes dye-bias correction, quantile normalization, and regression on correlated probe design bias adjustment. A total of 423 500 CpGs remained after quality control. DNA methylation values represent the proportion of methylated sites to the sum total of unmethylated (U) and methylated (M) sites at a given locus: β = M/(U + M + 100). We used logit-transformed β values (M values)^32^ in association analyses.

We calculated epigenetic age acceleration using 4 epigenetic clock measures developed by Horvath,^21^ Hannum et al,^22^ Levine et al^23^ (PhenoAge), and Lu et al^24^ (GrimAge). We used residuals from regressing chronologic age on epigenetic age to calculate epigenetic age acceleration; positive values signify that biological age is higher than chronologic age. For comparability of findings across clocks, we transformed epigenetic age metrics for each clock into *z* scores by subtracting the mean from each metric and dividing this difference by the SD.

### Statistical Analysis

We used linear regression to estimate associations between neighborhood deprivation and epigenetic age acceleration in crude models, models weighted for the case-cohort sampling scheme (weights calculated for cases as 1.31 and for noncases at the time of selection as 31.81), and weighted models adjusted for potential confounders selected a priori, including smoking status (current, former, or never), environmental smoke exposure at age 19 years or older age (yes or no), alcohol use (number of alcoholic drinks per week in the past 12 months; continuous), body mass index (BMI; continuous [calculated as weight in kilograms divided by height in meters squared]), annual household income (≤$49 000, $50 000-$99 999, $100 000-$200 000, or >$200 000), and educational level (high school/General Educational Development [GED] credential or less, some college/associate degree/technical degree, bachelor’s degree, or advanced degree). Models weighted for the case-cohort sampling scheme were estimated using the PROC SURVEYREG tool in SAS, version 9.4 (SAS Institute Inc). Annual household income was not thought to be on the causal path but may be associated with neighborhood SES. In sensitivity analyses, we tested the association between neighborhood deprivation and epigenetic age acceleration, additionally controlling for estimated proportions of CD4^+^ T cells, CD8^+^ T cells, monocytes, natural killer cells, and granulocytes. We excluded B cells to avoid model collinearity.

For epigenome-wide association study analyses, we used multivariable linear regression weighted for the sampling scheme using the R package survey to examine the association between neighborhood deprivation and DNA methylation at each of the 423 500 CpGs. To maximize power, neighborhood deprivation was modeled continuously as the independent variable. In addition to the aforementioned covariates, we also adjusted for top 10 surrogate variables estimated using nonnegative control probes and batched as previously described,^30^ chronologic age, and blood cell type proportions (CD8^+^ T cells, CD4^+^ T cells, natural killer cells, B cells, monocytes, or granulocytes) estimated using the Houseman et al^33^ method and Reinius et al^34^ reference panel. We used a threshold of false discovery rate–adjusted *P* value (*q* value) to correct for multiple testing.^35^ Results were considered statistically significant at *q* < .05 produced from a 2-sided hypothesis test. We also used a Bonferroni-adjusted *P* value as a cutoff for statistically significant associations, which is a more conservative method used to adjust for multiple testing (*P* = .05 / 423 500 CpGs = 1.18 × 10^−07^).

Secondary analyses explored associations between individual SES indicators, including annual household income and highest educational attainment, in association with epigenetic age acceleration in mutually adjusted models and adjusted for neighborhood deprivation as well as the other potential confounders to better understand the role of these factors independent of neighborhood traits. Statistical analyses were conducted in SAS, version 9.4 (SAS Institute) and R, version 3.6.2 (R Foundation for Statistical Computing).

## Results

Mean (SD) age of the participants was 56.9 (8.7) years, with a mean (SD) BMI of 27.7 (6.0). Overall, participants reported a mean (SD) of 3.1 (4.7) alcoholic drinks per week. A total of 1381 participants (52.5%) were never smokers and 1812 participants (68.9%) reported environmental tobacco smoke exposure. The most common category for annual household income was $50 000-$99 999 (1081 of 2630 [40.4%]) and for highest educational achievement was some college/associate or technical degree (842 [32.0%]). A total of 1497 women (56.9%) in the case cohort had developed breast cancer at the time of secondary data analysis. Only 589 study participants (22.4%) resided in a disadvantaged neighborhood (defined as having disadvantage >50th percentile of all US neighborhoods). Compared with individuals living in neighborhoods with lower deprivation scores (≤50th percentile in the US), those with greater neighborhood deprivation were more likely to be current smokers (56 [9.5%] vs 135 [6.6%]), have environmental tobacco smoke exposure (427 [72.5%] vs 1385 [67.9%]), achieved a high school education/GED or less (139 [23.6%] vs 264 [12.9%]), and have an annual household income less than or equal to $49 999 (246 [41.8%] vs 362 [17.7%]) (Table 1). Correlations between age acceleration *z* scores ranged from *r* = 0.13 to *r* = 0.57, with the lowest correlation between Horvath and GrimAge and the highest between Hannum and Horvath (eTable 1 in the Supplement).

**Table 1.** Participant Characteristics at Study Enrollment, Sister Study 2003-2009 (N = 2630)

Variable	Area deprivation, US percentile, No. (%)	
	>50th	≤50th
Total	589 (22.4)	2041 (77.6)
Age, mean (SD), y	57.8 (8.6)	56.6 (8.8)
Alcohol intake, mean (SD), drinks/wk	2.4 (4.5)	3.3 (4.8)
BMI	28.5 (6.0)	27.4 (6.0)
Breast cancer status at follow-up		
Event	254 (43.1)	879 (43.1)
Nonevent	335 (56.9)	1162 (56.9)
Smoking status		
Never	319 (54.2)	1062 (52.0)
Past	214 (36.3)	844 (41.4)
Current	56 (9.5)	135 (6.6)
Environmental tobacco smoke exposure		
No	162 (27.5)	656 (32.1)
Yes	427 (72.5)	1385 (67.9)
Educational level		
High school/GED or less	139 (23.6)	264 (12.9)
Some college/associate or technical degree	225 (38.2)	617 (30.2)
Bachelor's degree	114 (19.4)	595 (29.2)
Advanced degree	111 (18.8)	565 (27.7)
Income, household annual, $		
≤49 999	246 (41.8)	362 (17.7)
50 000-99 999	238 (40.4)	843 (41.3)
100 000-200 000	94 (15.9)	666 (32.6)
>200 000	11 (1.9)	170 (8.3)

Abbreviations: BMI, body mass index (calculated as weight in kilograms divided by height in meters squared); GED, General Educational Development.

In models weighted for the case-cohort sampling scheme, greater neighborhood deprivation (denoted by higher levels) was associated with increased age acceleration using all 4 clocks (Table 2). Among first-generation clocks, age acceleration was higher for the Hannum clock (level 4 vs level 1: β = 0.23; 95% CI, 0.01-0.45). No clear pattern of association was observed for the Horvath clock (level 4 vs level 1: β = 0.03; 95% CI, −0.20 to 0.26). Greater vs lower neighborhood deprivation showed positive associations with epigenetic age acceleration estimated by PhenoAge (level 4 vs level 1: β = 0.28; 95% CI, 0.06-0.50) and GrimAge (level 4 vs level 1: β = 0.37; 95% CI, 0.12-0.62). Associations with Hannum, PhenoAge, and GrimAge but not Horvath, exhibited a significant trend with increasing deprivation levels (*P* test for trend, Hannum: *P* = *.*001, PhenoAge: *P* < .001, and GrimAge: *P* < .001) (Table 2). Weighted models after adjusting for potential confounders were similar to unadjusted model results for the Horvath clock, but associations for other clocks were attenuated: Hannum clock (level 4 vs level 1: β = 0.09; 95% CI, −0.15 to 0.33) and PhenoAge (level 4 vs level 1: β = 0.16; 95% CI, −0.08 to 0.40). Similarly, associations with epigenetic age acceleration measured with GrimAge were no longer observed for the highest vs lowest deprivation score after adjusting for potential confounders (level 4 vs level 1: β = 0.12; 95% CI, −0.09 to 0.33) (Table 2). Models additionally controlling for estimated cell type proportions showed similar patterns of association both in magnitude and strength between neighborhood deprivation and epigenetic age acceleration across epigenetic clock metrics (eTable 2 in the Supplement).

**Table 2.** Associations Between Neighborhood Deprivation and Epigenetic Age Acceleration (N = 2630)

Variable	No. (%)	*z* Score								
		Horvath		Hannum		PhenoAge		GrimAge		
		β (95% CI)	*P* value	β (95% CI)	*P* value	β (95% CI)	*P* value	β (95% CI)	*P* value
**Crude models**										
Level 1: ≤25	1256 (47.8)	1 [Reference]		1 [Reference]		1 [Reference]		1 [Reference]	
Level 2: 26-50	785 (29.8)	0.06 (−0.03 to 0.15)	.21	0.11 (0.02-0.19)	.02	0.14 (0.05-0.23)	.003	0.18 (0.09-0.26)	.0001
Level 3: 51-75	430 (16.4)	0.06 (−0.05 to 0.17)	.28	0.13 (0.02-0.24)	.02	0.18 (0.07-0.29)	.001	0.26 (0.16- 0.37)	<.0001
Level 4: 76-100	159 (6.0)	0.01 (−0.15 to 0.18)	.86	0.16 (−0.01 to 0.32)	.06	0.17 (0.001-0.33)	.049	0.25 (0.09-0.42)	.002
**Weighted-unadjusted models**^**a**^										
Level 1: ≤25	1256 (47.8)	1 [Reference]		1 [Reference]		1 [Reference]		1 [Reference]	
Level 2: 26-50	785 (29.8)	0.11 (−0.01 to 0.23)	.08	0.20 (0.07-0.33)	.002	0.26 (0.14-0.39)	<.001	0.30 (0.17-0.42)	<.001
Level 3: 51-75	430 (16.4)	0.13 (−0.04 to 0.31)	.12	0.20 (0.04-0.36)	.01	0.35 (0.21-0.49)	<.001	0.35 (0.19-0.50)	<.001
Level 4: 76-100	159 (6.0)	0.03 (−0.20 to 0.26)	.83	0.23 (0.01-0.45)	.04	0.28 (0.06-0.50)	.01	0.37 (0.12-0.62)	.004
**Weighted and adjusted**^**a**^**^,^**^**b**^										
Level 1: ≤25	1256 (47.8)	1 [Reference]		1 [Reference]		1 [Reference]		1 [Reference]	
Level 2: 26-50	785 (29.8)	0.09 (−0.04 to 0.23)	.17	0.14 (0.002-0.28)	.047	0.21 (0.08-0.34)	.001	0.16 (0.06-0.27)	.003
Level 3: 51-75	430 (16.4)	0.12 (−0.06 to 0.31)	.19	0.12 (−0.05 to 0.30)	.17	0.29 (0.13-0.45)	<.001	0.23 (0.10-0.37)	<.001
Level 4: 76-100	159 (6.0)	−0.01 (−0.27 to 0.24)	.93	0.09 (−0.15 to 0.33)	.45	0.16 (−0.08 to 0.40)	.20	0.12 (−0.09 to 0.33)	.26

^a^ Weighted for case-cohort sampling scheme.

^b^ Adjusted for smoking status, environmental tobacco smoke, alcohol, body mass index, income, and educational level.

Epigenome-wide analyses identified 3 false discovery rate–significant (*q* < .05) differentially methylated loci associated with neighborhood deprivation: cg23538773 (no known gene; β = 0.002352, *q* = .01), cg07390373 (*MAOB*; β = 0.001854, *q* = .02), cg18956825 (no known gene; β = −0.00233, *q*=.04) (Table 3, Figure). Greater neighborhood deprivation was associated with higher methylation levels at the cg07390373 loci (β = 0.001854; *P* = 9.71 × 10^−08^) and cg23538773 loci (β = 0.002352; *P* = 1.98 × 10^−08^). However, greater neighborhood deprivation was associated with lower methylation levels at cg18956825 (β = −0.00233; *P* = 2.75 × 10^−07^). The first 2 associations were statistically significant even after Bonferroni correction.

**Table 3.** Top 20 Statistically Significant CpG Sites in EWAS of Neighborhood Deprivation, Sister Study 2003-2009 (N = 2621)^a^

Probe	CHR	Position^**b**^	Gene	β	*P* value	*q* value
cg23538773	14	89493162		0.002352	1.98 × 10^−08^	.01
cg07390373	X	43741933	*MAOB*	0.001854	9.71 × 10^−08^	.02
cg18956825	7	63363033		−0.00233	2.75 × 10^−07^	.04
cg27493484	17	37793215	*STARD3*	−0.00143	7.00 × 10^−07^	.07
cg13585240	3	48959375	*ARIH2OS*	−0.00118	1.39 × 10^−06^	.11
cg05943563	16	88636557	*ZC3H18*	−0.00185	2.08 × 10^−06^	.14
cg21158178	X	54070874	*PHF8*	0.001565	2.86 × 10^−6^	.16
cg00644033	7	100544704		−0.00140	3.15 × 10^−06^	.16
cg12695487	3	151625374		−0.00101	4.22 × 10^−06^	.19
cg23534593	X	47342469	*ZNF41*	0.001565	5.12 × 10^−06^	.20
cg13208102	X	102611412	*WBP5*	0.002660	5.73 × 10^−06^	.21
cg16443970	10	102504022	*PAX2*	−0.00082	7.57 × 10^−06^	.23
cg06044468	8	104177564	*BAALC*	−0.00111	7.65 × 10^−06^	.23
cg27107094	22	37768915	*ELFN2*	−0.00137	8.20 × 10^−06^	.23
cg01551258	17	30479187	*ARGFXP2*	0.001554	9.62 × 10^−06^	.26
cg25862768	8	145578141	*TMEM249*	0.001822	1.08 × 10^−05^	.27
cg04384208	1	161519396	*FCGR3A*	0.001839	1.23 × 10^−05^	.29
cg26614815	12	14996776	*ART4*	−0.00096	1.87 × 10^−05^	.39
cg17347941	15	79387269	*RASGRF1*	−0.00298	1.98 × 10^−05^	.39
cg19923333	5	131831966		−0.00107	2.00 × 10^−05^	.39

Abbreviations: CHR, chromosome; CpG, cytosine-phosphate-guanine; EWAS, epigenome-wide association study.

^a^ Model adjusted for blood cell type proportions, 10 control surrogate variables, plate, and age at baseline; adjusted for sample weight, smoking status, environmental tobacco smoke exposure, body mass index, income, educational level, and alcohol use; and weighted for case-cohort sampling scheme. Nine participants did not have covariate information and are not included.

^b^ Genome build hg19.

**Figure.**
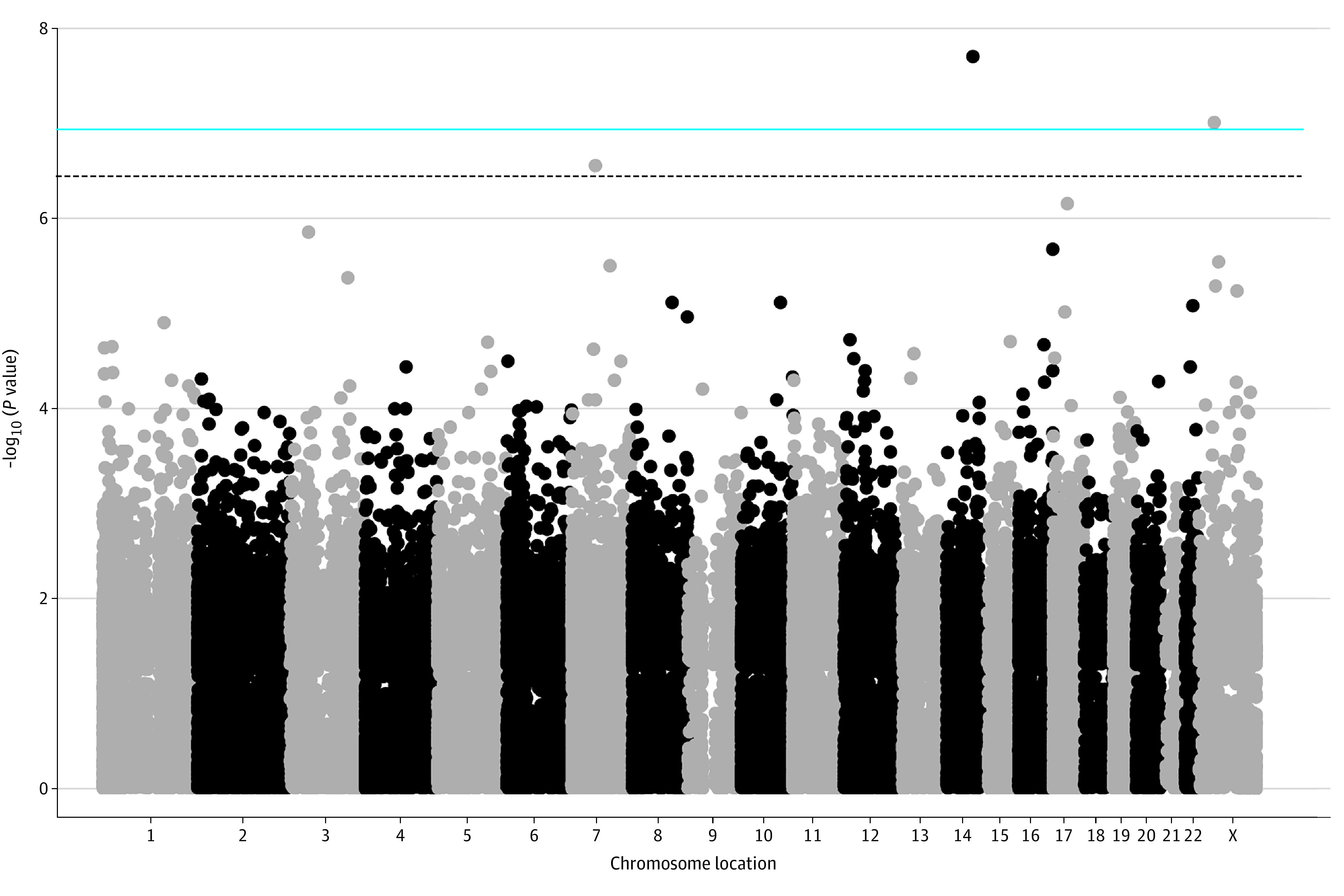
Manhattan Plot for Epigenome-Wide Association With Area Deprivation Index

In analyses of individual-level SES indicators, annual household income showed no associations with epigenetic age acceleration across all 4 clocks. Educational attainment was also not associated with epigenetic age acceleration measured by Horvath, PhenoAge, and GrimAge. However, using the Hannum clock and GrimAge, participants with some college/associate/technical degree had higher epigenetic age acceleration for Hannum (β = 0.19; 95% CI, 0.05-0.33) compared with those with a bachelor’s degree (Table 4).

**Table 4.** Individual Socioeconomic Status and Epigenetic Age Acceleration Among Sister Study Participants 2003-2009 (N = 2630)^a^

Variable	No. (%)	Clock							
		Horvath		Hannum		PhenoAge		GrimAge (*z* score)	
		β (95%CI)	*P* value	β (95%CI)	*P* value	β (95%CI)	*P* value	β (95%CI)	*P* value
Income, $									
<49 999	608 (23.1)	−0.11 (−0.37 to 0.14)	.39	0.14 (−0.09 to 0.37)	.25	0.005 (−0.24 to 0.25)	.97	0.10 (−0.09 to 0.29)	.30
50 000-99 999	1081 (41.1)	−0.08 (−0.30 to 0.15)	.51	0.03 (−0.17 to 0.22)	.78	−0.05 (−0.26 to 0.16)	.63	0.04 (−0.12 to 0.20)	.64
100 000-200 000	760 (28.9)	−0.08 (−0.31 to 0.15)	.51	−0.09 (−0.29 to 0.10)	.35	−0.12 (−0.34 to 0.09)	.26	0.0003 (−0.16 to 0.16)	.99
>200 000	181 (6.9)	1 [Reference]		1 [Reference]		1 [Reference]		1 [Reference]	
Educational level									
≤GED or completion of high school	403 (15.3)	0.02 (−0.16 to 0.21)	.81	0.10 (−0.09 to 0.29)	.28	0.01 (−0.17 to 0.19)	.93	0.11 (−0.04 to 0.26)	.13
Some college/associate/technical degree	842 (32.0)	0.07 (−0.07 to 0.21)	.34	0.19 (0.05-0.33)	.009	0.05 (−0.08 to 0.19)	.45	0.08 (−0.03 to 0.20)	.16
Bachelor's degree	709 (27.0)	1 [Reference]		1 [Reference]		1 [Reference]		1 [Reference]	
Master's or doctoral degree	676 (25.7)	0.04 (−0.12 to 0.20)	.60	0.14 (−0.01 to 0.30)	.07	0.06 (−0.08 to 0.20)	.42	−0.09 (−0.21 to 0.03)	.14

Abbreviation: GED, General Educational Development.

^a^ Weighted for case-cohort sampling scheme. Adjusted for smoking status, environmental tobacco smoke, alcohol, and body mass index, and mutually adjusted for socioeconomic status indicator factors (income, educational level, and Area Deprivation Index level).

## Discussion

Within our case-cohort sample, contemporaneous neighborhood deprivation appeared to be associated with epigenetic age acceleration. Specifically, we observed that individuals with the highest vs lowest levels of neighborhood deprivation had higher epigenetic age acceleration using Hannum, PhenoAge, and GrimAge but not Horvath clocks. This finding supports previous research^36^ suggesting that living in stressful environments leads to adverse biological responses and increased aging. Among the individual-level SES factors examined, we found that participants with some college-level education had accelerated age compared with those with a bachelor’s degree (specifically with the Hannum clock) but found no associations with annual household income.

Differences in results by methods suggest that detection of associations depends on the clock. First-generation clocks published by Horvath (353 CpGs) and Hannum (71 CpGs) were derived from chronologic age alone, whereas second-generation clocks PhenoAge (513 CpGs) and GrimAge (1030 CpGs) include a larger representation of CpGs that incorporate phenotypic information. For example, GrimAge incorporates behavioral phenotypes (eg, DNAm surrogates of pack-years) not considered in the construction of other clocks. Because the GrimAge clock incorporates DNAm surrogates of several mortality-associated biomarkers, it appears to be the most sensitive to the biological outcomes associated with living in a disadvantaged neighborhood.

In addition to observing differences in associations with different clocks, we also noted variations in associations before and after the inclusion of covariates. After consideration of additional covariates, associations attenuated across clock metrics. It is possible that additional model adjustments induced overadjustment bias by incorporating mediators on the causal path,^37^ and it is for this reason that we chose a priori to interpret unadjusted models for the main analysis. Effect estimates of adjusted associations between neighborhood deprivation and GrimAge appeared to be the most affected by attenuation, possibly owing to the additional incorporation of phenotypic information in clock construction as previously mentioned.

Observed associations between neighborhood deprivation and epigenetic clocks agree with the previous report of this association.^38^ Among a group of African American women (N = 100), higher neighborhood deprivation was found to be linked to higher epigenetic age acceleration measured by the Hannum clock. African American women may face higher exposure to neighborhood deprivation as well as exposure to other area-level stressors (eg, racial discrimination) that may affect epigenetic factors.^39^ While our study was restricted to non-Hispanic white women, agreement with this previous work suggests that the presence of a link between neighborhood and epigenetic age acceleration may be robust across different populations.

Existing studies on individual-level SES and epigenetic age acceleration lend additional support to our findings. Hughes et al^40^ assessed a cohort of adults living in the UK (N = 1099) and found that participants who were more disadvantaged in childhood had increased epigenetic age measured using the Horvath and Hannum clocks. Using the PhenoAge metrics, Liu et al^41^ reported that less educated postmenopausal women had higher epigenetic age than other women.

We saw some evidence of higher epigenetic age acceleration associated with lower educational levels using the Hannum clock. Other studies have found that higher educational level and healthy diet are weakly associated with lower epigenetic aging according to the Hannum,^22,42^ PhenoAge,^43^ and GrimAge^24^ clocks. These findings provide further support that epigenetic age metrics are promising biomarkers for socioeconomic stressors. Furthermore, race/ethnicity and sex are associated with epigenetic aging.^44^ Previous work has also shown that women age more slowly than men^44^; thus, our findings cannot be extrapolated to a male population. In addition, Hispanic ancestry has been associated with slower epigenetic aging than European ancestry according to the Horvath pan tissue clock^21^ but not according to the Hannum clock.^22^ Interpretation of associations between markers of SES and epigenetic age metrics may need to be considered in the context of ancestral differences.

In epigenome-wide association study analyses, we identified 3 loci associated with neighborhood deprivation: cg23538773, cg07390373, and cg18956825. One of the CpG sites identified is annotated to *MAOB*, which has been previously related to Parkinson disease, a prominent age-related disease.^45,46^ Previous studies have explored methylation in association with neighborhood deprivation.^18,47,48,49^ However, none of the CpGs that we identified overlapped with CpGs identified in these earlier studies.

To our knowledge, this study is one of the first to assess the association between neighborhood disadvantage and epigenetic age acceleration metrics. Our findings suggest that there may be a biologically measurable consequence of living in a neighborhood with deprivation. Other primary study strengths include the assessment of residential addresses at the time of the blood draw, which may best suit blood-based DNA methylation, for which malleable changes over time have been observed.^50^ We also used 4 different established methods to estimate epigenetic age acceleration; to our knowledge, 3 of these have never been studied in relation to neighborhood disadvantage. In our study, the association between neighborhood deprivation and epigenetic age acceleration was inconsistent across clocks. We observed associations between neighborhood deprivation and epigenetic age acceleration with Hannum, PhenoAge, and GrimAge but not Horvath metrics. Differences in results by methods suggest that detection of associations depends on clock methods, which is important given the absence of a commonly used standardized clock method in the literature. In our study, we leveraged an established measure of deprivation that is publicly available and enumerates deprivation relative to all neighborhoods across the US. Benefits of using this index include the potential future use in other cohorts for comparability/standardization across study results and the small area spatial resolution at the census block-group level. In addition, our study considered individual SES factors, such as income and educational level, allowing us to identify independent neighborhood effects.

### Limitations

Limitations of our study include lack of generalizability because the sample was restricted to non-Hispanic White women. Health disparities associated with SES may be stronger among Black individuals and other racial and ethnic minorities compared with White individuals in the US.^51^ Thus, the results may underestimate the magnitude of neighborhood deprivation-related age acceleration present in other groups, particularly among those of races/ethnicities other than White. Similarly, men may be more vulnerable to SES-related mortality compared with women.^52^ A primary limitation of our study is its cross-sectional design. Aging is a complex process that unfolds over time. However, our analysis does not account for long-term exposure to neighborhood deprivation—only current exposure was examined. Individuals likely experience a range of neighborhoods over their life course. However, the ADI metric that we used is measured only since 2000, so we were unable to use this metric to characterize prior residences among the study participants. Furthermore, exposure to neighborhood deprivation during certain developmental periods may make individuals more susceptible to accelerated aging. It has been suggested that early epigenetic modifications may be maintained over the life course.^40^ One study described the relative importance of childhood SES on epigenetic aging compared with that of adulthood. However, we expect any within-person error related to neighborhood deprivation over time to be nondifferential by epigenetic age status. We also expect any bias to be lessened by the residential stability of Sister Study participants since 54% of the total cohort reported at baseline that they were living in the residence they had lived in the longest since age 20 years.

Additional limitations include a cautious interpretation of epigenetic clocks.^53^ Epigenetic clocks only mark or estimate age and are not necessarily a part of the aging process. In addition, genetic variation within our study population may play a role in clock measures. A final limitation is that our findings for epigenetic signatures of neighborhood deprivation require confirmation in other populations.

## Conclusions

The findings of this large study provide possible evidence of the association between neighborhood deprivation and epigenetic age acceleration. Results contribute to a growing body of knowledge of biological markers of age and the role that DNA methylation may play in the association between neighborhood characteristics and age-related disease. Our findings support the Hannum, PhenoAge, and GrimAge clocks as sensitive markers of neighborhood deprivation. Further research is needed to better understand mechanisms explaining the association between neighborhood deprivation and epigenetic age.

## Notes

Supplement. **eTable 1.** Correlations Between Epigenetic Age Acceleration Metrics **eTable 2.** Associations Between Neighborhood Deprivation and Epigenetic Age Acceleration (N = 2,630), Accounting for Cell-Type Proportions
