# Supplementary material for: Association of Neighborhood Deprivation With Epigenetic Aging Using 4 Clock Metrics
Source: JAMA Netw Open. 2020 Nov 4;3(11):e2024329. doi: 10.1001/jamanetworkopen.2020.24329 (PMC7643028; doi:10.1001/jamanetworkopen.2020.24329)
Supplement: Supplement. — eTable 1. Correlations Between Epigenetic Age Acceleration Metrics eTable 2. Associations Between Neighborhood Deprivation and Epigenetic Age Acceleration (N = 2,630), Accounting for Cell-Type Proportions [file jamanetwopen-e2024329-s001.pdf]

Supplementary Online Content

Lawrence KG, Kresovich JK, O'Brien KM, et al. Association of neighborhood deprivation with epigenetic aging using 4 clock metrics. *JAMA Netw Open*. 2020;3(11):e2024329. doi:10.1001/jamanetworkopen.2020.24329

eTable 1. Correlations Between Epigenetic Age Acceleration Metrics

eTable 2. Associations Between Neighborhood Deprivation and Epigenetic Age Acceleration (N=2,630), Accounting for Cell-Type Proportions

This supplementary material has been provided by the authors to give readers additional information about their work.

© 2020 Lawrence KG et al. *JAMA Network Open.*

**eTable 1. Correlations between epigenetic age acceleration metrics**				
	Horvath	Hannum	Phenoage	GrimAge
Horvath	1.00			
Hannum	0.57	1.00		
Phenoage	0.49	0.48	1.00	
Grimage	0.13	0.25	0.44	1.00

© 2020 Lawrence KG et al. *JAMA Network Open.*

**eTable 2. Associations between neighborhood deprivation and epigenetic age acceleration (N=2,630), accounting for cell-type proportions[a]**									
		Horvath (z-score)		Hannum (z-score)		PhenoAge (z-score)		GrimAge (z-score)	
Model 1									
	N(%)	β(95%CI)	p-value	β(95%CI)	p-value	β(95%CI)	p-value	β(95%CI)	p-value
Level 1: ≤25	1256(47.8)	Ref	Ref	Ref	Ref	Ref	Ref	Ref	Ref
Level 2: 26-50	785(29.8)	0.10(-0.02,0.22)	0.10	0.17(0.05,0.29)	0.006	0.24(0.13,0.36)	<.0001	0.28(0.16,0.40)	<.0001
Level 3: 51-75	430(16.4)	0.10(-0.07,0.27)	0.24	0.09(-0.06,0.24)	0.22	0.26(0.12,0.40)	0.0002	0.31(0.16,0.46)	<.0001
Level 4: 76-100	159(6.0)	-0.01(-0.23,0.22)	0.94	0.14(-0.07,0.34)	0.20	0.23(0.02,0.44)	0.03	0.38(0.12,0.63)	0.004
Model 2									
	N(%)	β(95%CI)	p-value	β(95%CI)	p-value	β(95%CI)	p-value	β(95%CI)	p-value
Level 1: ≤25	1256(47.8)	Ref	Ref	Ref	Ref	Ref	Ref	Ref	Ref
Level 2: 26-50	785(29.8)	0.09(-0.04, 0.23)	0.18	0.12(-0.01,0.25)	0.07	0.20(0.08,0.32)	0.001	0.15(0.05,0.25)	0.004
Level 3: 51-75	430(16.4)	0.10(-0.08, 0.29)	0.27	0.04(-0.12,0.21)	0.60	0.22(0.07,0.37)	0.005	0.18(0.06,0.31)	0.005
Level 4: 76-100	159(6.0)	-0.03(-0.28, 0.22)	0.82	0.03(-0.19, 0.26)	0.78	0.13(-0.09,0.36)	0.25	0.12(-0.08,0.33)	0.23

[a]Weighted for case-cohort sampling scheme

Model 1: Houseman cell type proportions (CD4+ T-cells, CD8+ T-cells, monocytes, natural killer cells, and granulocytes)

Model 2: Model 1+ smoking status, environmental tobacco smoke, alcohol, BMI, income, education

© 2020 Lawrence KG et al. *JAMA Network Open.*
